# From a stem-cell–centered to a niche-centered view: the core role of collagen networks in hair loss and hair follicle miniaturization

**DOI:** 10.3389/fcell.2026.1824126

**Published:** 2026-05-29

**Authors:** Zhounan Jiang, Ye Xu, Yu Lou

**Affiliations:** 1 College of Basic Medical Sciences, Zhejiang Chinese Medical University, Hangzhou, China; 2 Medical Cosmetic Center, Affiliated Hangzhou First People’s Hospital, Westlake University School of Medicine, Hangzhou, China

**Keywords:** androgenetic alopecia, collagen network, extracellular matrix remodeling, hair follicle miniaturization, stem cell niche

## Abstract

Hair follicle miniaturization is a quantifiable histopathological endpoint shared by multiple forms of alopecia. The conventional “stem cell–centric” view often attributes regenerative failure to depletion or intrinsic dysfunction of hair follicle stem cells (HFSCs). However, in canonical trajectories such as human androgenetic alopecia, HFSC-related populations may remain detectable by marker-based analyses, whereas progenitor output is reduced. This pattern suggests that impaired conversion from quiescent HFSCs into an expandable progenitor/transit-amplifying compartment may contribute to miniaturization, while not excluding concomitant HFSC functional decline. We therefore propose “niche identity,” which treats the follicular niche as a set of measurable, stratifiable, and intervention-amenable structural–mechanical constraints. We posit that the collagen network may act as an integrative hub that influences regenerative thresholds and the stability of lineage output through interfacial continuity, fibrillar topology, and local mechanical states. Niche identity is defined here by five coupled state variables: basement membrane boundary integrity, adhesion/anchoring apparatuses, fibrillar topological organization, mechanical set-points, and hair cycle–scaled dynamic remodeling windows. We propose that these elements may drift coordinately under androgen-biased profibrotic remodeling, chronic low-grade inflammation with MMP-mediated matrix degradation, and aging/glycation-associated crosslinking and stiffening, thereby locking follicles into a low-output steady state. Finally, we discuss “signal–structure mismatch” as a plausible basis for unstable therapeutic responses and relapse and propose a niche identity–oriented translational framework intended to guide future experimental testing and endpoint selection.

## 1 Introduction

Hair follicle miniaturization represents one of the most quantifiable histological endpoints across various hair loss conditions (Kamishima et al., 2024; Kasumagic-Halilovic, 2021). Its hallmark manifestations include a leftward shift in hair shaft diameter distribution toward finer calibers, an altered terminal-to-vellus-like hair ratio, and an overall reduction in the physical dimensions of the follicle, which are typically accompanied by a decreased anagen-to-telogen ratio and aberrant cycle dynamics (Altendorf et al., 2026). However, clinical “alopecia” is not universally driven by miniaturization as its core pathology. For instance, telogen effluvium is predominantly characterized by altered cycle ratios and shedding dynamics, without necessarily exhibiting a systemic phenotypic shift from terminal to vellus-like hairs (Rebora, 2019). Conversely, cicatricial (scarring) alopecias are primarily driven by immune-mediated destruction and subsequent fibrosis, culminating in irreversible structural loss (Karnik et al., 2009). To prevent the conflation of phenotypic endpoints with underlying mechanisms, this review focuses primarily on the miniaturization spectrum (typified by androgenetic alopecia, AGA), while utilizing other subtypes as boundary conditions to delineate the framework’s applicability.

The traditional stem cell–centered explanation often equates regenerative failure directly with depletion or intrinsic dysfunction of hair follicle stem cells (HFSCs). However, in typical miniaturizing alopecia, HFSC-related populations can remain detectable by marker-based analyses, while progenitor-associated compartments are reduced or transcriptionally shifted (Garza et al., 2011; Rathman-Josserand et al., 2013; Charoensuksira et al., 2025; Jang et al., 2023). This observation should be interpreted as structural or phenotypic retention rather than proof of normal HFSC function. Surface-marker detectability does not exclude functional exhaustion, epigenetic reprogramming, or reduced responsiveness to activation cues. Therefore, the central issue is not whether HFSCs are intrinsically normal or externally constrained, but how cell-intrinsic decline and niche-derived structural constraints interact to limit progenitor production and regenerative output.

The stem cell niche should not be relegated to an amorphous “microenvironment”; instead, it represents a defined set of measurable and therapeutically targetable physical and biochemical constraints (Zhang and Chen, 2024). Herein, we introduce the concept of “Niche Identity” to conceptualize the niche as a collection of quantifiable parameters, highlighting the collagen network as its central organizing hub (Adam et al., 2020). Serving as the primary structural scaffold for both the basement membrane (BM) and the dermal extracellular matrix (ECM), the collagen system regulates interfacial continuity, topological organization, and local material mechanics (Khalilgharibi and Mao, 2021; Vining and Mooney, 2017). Alterations in these structural properties can chronically dictate the efficiency and stability of lineage output, independent of shifts in single upstream signaling cascades (Wang et al., 2023). Under this framework, the critical variable driving the miniaturization spectrum is not the absolute abundance of collagen, but rather the spatiotemporal and mechanical deviations within the network’s organization (Wang et al., 2024). As these structural aberrations accumulate, the supply of progenitor cells is chronically throttled despite the physical retention of HFSCs, ultimately manifesting as the quantifiable endpoint of miniaturization (Jang et al., 2023).

The five-element framework is not intended to relabel known niche features as a new taxonomy. Its explanatory value lies in treating boundary integrity, anchoring, fibrillar topology, mechanics, and remodeling dynamics as coupled state variables rather than independent lesions. Single-element models can explain local effects, such as impaired HFSC activation after niche stiffening or anchoring instability after COL17A1/hemidesmosome loss. The coupled model makes a different prediction: durable regeneration should require matched restoration of interfacial continuity and matrix reset capacity. Thus, matrix softening alone should have limited durability if BM/COL17A1-dependent anchoring remains discontinuous, whereas preserved anchoring should not sustain anagen if the surrounding fibrillar matrix remains topologically constricted, highly crosslinked, or slow-relaxing. A signal-only or single-element intervention is therefore predicted to produce transient output in a structurally mismatched niche, whereas combined restoration of anchoring continuity and mechanical/remodeling permissiveness should better preserve progenitor expansion, follicle depth, and shaft caliber after withdrawal of pro-growth stimulation.

To rigorously distinguish causality from mere correlation, this review demands evidence rooted in co-localized, multimodal registration and targeted spatial manipulation (He G. et al., 2023). Specifically, structural and biomechanical readouts must spatially align with progenitor deficits, and the necessity and/or sufficiency of key variables in resetting the regenerative threshold must be validated under controlled conditions (Koester et al., 2021; Wei et al., 2023). Guided by this rigorous epistemological framework, subsequent sections will integrate pathophysiological mechanisms and propose stratified translational pathways.

## 2 Structural–mechanical dimensions of niche identity: collagen networks as an integrative hub

**FIGURE 1:**
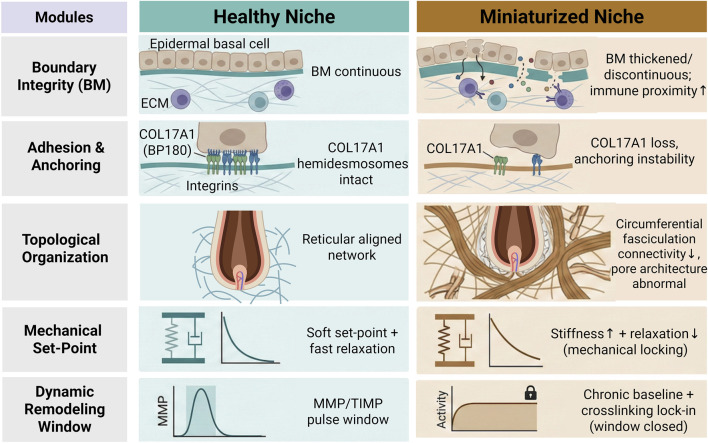
Healthy vs. Miniaturized niche, Five elements. Boundary integrity, anchoring (COL17A1/hemidesmosomes), topology, mechanics (stiffness ↑, relaxation ↓), and remodeling dynamics (MMP/TIMP window loss) shift from a stable, aligned, compliant niche to a leaky, weakly anchored, fasciculated, mechanically locked, chronically remodeled state, resulting in HFSC persistence but insufficient progenitor supply, superficialization, and shaft thinning. Abbreviations: AGA, androgenetic alopecia; HFSC, hair follicle stem cell; MMP, matrix metalloproteinase; TIMP, tissue inhibitor of metalloproteinases.

Stem cell behavior is subjected to non-cell-autonomous regulation by the niche. The niche’s capacity to “calibrate stem cell output” across diverse physiological states and external perturbations generally does not rely on a single signaling pathway, but rather on a constellation of structural and mechanical conditions harbored within the ECM (Fujiwara, 2024; Estrach et al., 2025). In the context of the hair follicle, these conditions are organized and transduced primarily through the collagen network. Ranging from BM-associated collagens that maintain compartmental boundaries, to anchoring collagens that stabilize cell-matrix junctions, and fibrillar collagens that dictate topological connectivity and tensional/viscoelastic set-points, the collagen network constrains HFSC localization, division, and lineage output (Figure 1) (Liu Y. et al., 2022; Morinaga et al., 2021). While cyclical hair growth is driven by HFSCs, the stable progression of the hair cycle, and its pathological diversion toward a “miniaturized, low-output steady state” in alopecia, is fundamentally dictated by the collagen network-defined niche identity (Charoensuksira et al., 2025). Here, “coupling” refers to a conditional dependency among interface, topology, mechanics, and remodeling kinetics: the biological effect of one variable depends on the state of the others. Reduced stiffness is predicted to be regenerative only when interfacial anchoring and matrix remodeling capacity remain sufficient to support cell retention, polarity, and follicular downgrowth. Likewise, preserved COL17A1-associated anchoring is predicted to be insufficient if the surrounding fibrillar matrix remains mechanically locked or topologically non-permissive. This conditional logic distinguishes the framework from models that attribute impaired output to stiffness, anchoring, inflammation, or collagen load alone.

This review was selected to integrate evidence from hair follicle biology, extracellular matrix biology, mechanobiology, fibrosis, aging, and alopecia pathology. No predefined database-wide search string, formal screening workflow, or fixed inclusion/exclusion algorithm was applied. Therefore, the review does not claim exhaustive coverage of all available studies, and contradictory or negative findings may be underrepresented. To avoid overinterpretation, we distinguish, where relevant, among human observational evidence, murine interventional evidence, engineered matrix systems, and conceptual extrapolations.

### 2.1 Five elements of niche identity

#### 2.1.1 Boundary integrity

The functional niche intrinsically requires distinct physical demarcations to achieve compartmentalization. Confining stem cells to specific anatomical sites (e.g., the bulge) and maintaining a relatively stable, immune-privileged microenvironment amidst systemic inflammatory fluctuations is essential for preserving stem cell quiescence and an undifferentiated state (Li and Tumbar, 2021; Rousselle et al., 2022; Liu Z. et al., 2022; Suzuki et al., 2024). In the hair follicle, this boundary is primarily constituted by the BM. Enriched in key components such as Laminin-511/332 and Type IV collagen (COL4), the BM couples with cell-matrix adhesion/hemidesmosome complexes to form a stable interface (Has and Nystrom, 2015; Te Molder et al., 2021). This dense interface not only mitigates the risk of cell emigration but also acts as a selective barrier via diffusional resistance and ligand-binding kinetics, thereby restricting the influx of pro-inflammatory or pro-differentiating signals into the niche core and resetting local activation thresholds. Boundary integrity is not a static barrier; its thickness, continuity, and lamination undergo reversible remodeling in phase with the hair cycle. Once this renewal process skews toward a pathological steady state, compartmentalization can be chronically compromised (Koester et al., 2021; An et al., 2025). Miniaturized follicles frequently exhibit discontinuous, ruptured, or pathologically thickened BMs, indicative of degraded compartmentalization and signaling isolation failure (Li et al., 2022). A testable consequence of boundary failure is that in miniaturized regions, pro-inflammatory or pro-differentiating signals are more likely to spatially overlap with the HFSC neighborhood, co-occurring locally with anchoring instability and restricted lineage output. Conversely, if the BM structure and interfacial anchoring remain intact, the niche may sustain high regenerative capacity despite systemic signal fluctuations (Choi et al., 2021).

#### 2.1.2 Adhesion and anchoring

Stable niche identity requires that stem cells be physically retained through specific receptor–ligand interactions. This anchorage is not only positional but also instructive: adhesion complexes couple the cell to ECM, providing survival inputs and gating state transitions. In hair follicles, the core anchoring module is the integrin–BM axis, particularly α6β4 and β1-associated integrins that bind BM and form hemidesmosomes to achieve high-strength, long-time-scale adhesion (Nystrom and Kiritsi, 2021; Wenta et al., 2022). Hemidesmosomes mechanically couple the cytoskeleton to the BM, furnishing bulge HFSCs with a stable interface and a directional load-bearing context; together with compartment boundaries, they constitute an integrated boundary–anchorage niche interface (Roig-Rosello et al., 2024). Functionally, anchorage supports anoikis resistance and provides hair-cycle gating: stronger adhesion in telogen favors quiescence, whereas regeneration initiation requires reversible adjustment (a controlled decrease in adhesion) to permit migration, expansion, and lineage output (Nayak and Ge, 2025). Thus, anchorage operates as a time-dependent threshold control: overly strong adhesion can impede activation dynamics, whereas overly weak adhesion increases positional loss and survival risk (Estrach et al., 2025). Mechanistically, downregulation of adhesion molecules or hemidesmosome destabilization can act as an early signal of departure from niche constraints. A testable prediction is that, in advancing miniaturization, anchorage abnormalities will co-occur with boundary compromise and spatially align with the pattern “HFSC detectable but progenitor supply insufficient.” Failure modes may split: premature loosening predisposes to positional drift, whereas failure to reset anchorage cyclically predisposes to impaired regeneration initiation (Isogai et al., 2025).

#### 2.1.3 Topological organization

The topology of the stem cell niche refers to the spatial organization and geometric constraints of the ECM fibrillar network, encompassing fiber alignment (anisotropy), connectivity, pore size, density gradients, inter-layer coupling, and nanoscale surface topography (Niraula et al., 2025; Skillin et al., 2024; Da Silva Andre and Labouesse, 2024). For a cell, these topological variables define the geometric boundary conditions: the pore dimensions available for spreading, the permissible directions for establishing polarity and traction fibers, and the diffusibility and accessibility of signaling molecules within the network, thereby governing how local forces are propagated, concentrated, or dissipated (Peng et al., 2025). In miniaturizing alopecia, the decline in regenerative output reflects not only cellular signaling alterations but also a systemic shift in the geometric constraints imposed by the ECM fibrillar network. Within the follicular microenvironment, collagen fibers surrounding the dermal sheath and dermal papilla (DP) typically exhibit measurable anisotropic alignment or relatively stable reticular architectures. These structures provide geometric and mechanical boundary conditions essential for establishing cellular polarity, biasing division planes, and directing migration, thereby facilitating the orderly axial progression of lineage output (Skillin et al., 2024). Under the chronic drive of androgenic signaling, low-grade inflammation/oxidative stress, and the concomitant altered states of fibroblasts and ECM remodeling bias, the collagen network becomes highly susceptible to decreased alignment ordering, simplified connectivity, and aberrant pore/gradient structures. These alterations rewrite the geometric constraints, diminishing the spatial continuity required for progenitor cells to rebuild deeper follicular structures. The deviation of topological parameters spatially co-localizes with progenitor deficits and reduced follicle depth/volume, worsening progressively along a gradient correlated with disease stage. Furthermore, this topological shift aligns with local inflammatory or fibrotic readouts, indicating that structural rearrangement is not merely a coincidental byproduct, but is mechanistically coupled with the ECM remodeling bias induced by pathogenic inputs (Li L. et al., 2025).

#### 2.1.4 Mechanical set-point

The persistent state of restricted regenerative output in miniaturizing alopecia is intimately linked to an elevated mechanical threshold within the niche (Elosegui-Artola et al., 2023). The “mechanical set-point” is defined as the homeostatic range of the local biomechanical microenvironment, typically characterized by elasticity (e.g., Young’s modulus, kPa), and must be contextualized alongside viscoelasticity and tissue tension (Charbonier et al., 2025). The response of follicular cells to mechanical inputs is not solely dictated by instantaneous stiffness; under identical elastic conditions, divergent viscoelastic parameters and tensional backgrounds can profoundly alter the effective mechanical input, thereby resetting mechanotransduction thresholds and altering fate decision probabilities (Narasimhan and Fraley, 2025). Regenerative competence is determined not by stiffness alone, but by whether cells can complete effective mechanical remodeling within the relevant biological timescale. Accordingly, the stress-relaxation time constant, rather than elastic modulus alone, is one of the key parameters defining whether a niche supports lineage progression and regenerative maintenance. Recent work further shows that, in three-dimensional matrices, cell fate does not follow a simple linear rule of “the stiffer, the stronger.” Instead, it depends on whether the matrix permits sustained cell–matrix remodeling: stiffness provides the boundaries for traction and load bearing, whereas stress relaxation and viscoelasticity determine whether those traction forces can be converted into effective mechanical work over biologically relevant timescales, thereby further engaging mechanotransduction programs involving YAP/TAZ, PI3K–AKT, and epigenetic regulation (Kersey et al., 2024).

Scalp tissue afflicted by miniaturization frequently exhibits hardening or fibrotic tendencies coupled with altered viscoelastic profiles, indicating that the set-point has drifted away from the “pro-regenerative” optimal range. Cells translate this biomechanical drift into transcriptional reprogramming via mechanotransduction pathways (such as YAP/TAZ and their upstream adhesion-cytoskeleton coupling mechanisms): an excessively stiff set-point or viscoelastic imbalance can elevate the threshold for sustaining regeneration and increase the propensity for differentiation bias or senescence-associated programs (Peng et al., 2025). Consequently, regeneration becomes increasingly prone to a phenotype where anagen is initiated but fails to be sustained. This quantifiable drift in the mechanical set-point exhibits a stage-dependent correlation with progenitor insufficiency and miniaturization indices in the affected regions, transcending mere alterations in gross collagen content (Table 1).

#### 2.1.5 Dynamic remodeling

The relentless progression of miniaturizing alopecia signifies a transition of niche remodeling from cyclical renewal to a pathologically biased steady state. “Dynamic remodeling” denotes the relative rates and temporal window configurations of ECM synthesis, degradation, and crosslinking across the timescale of the hair cycle (Mayorca-Guiliani et al., 2025; Lloyd and He, 2024). The mechanistic crux lies not in whether remodeling occurs, but whether a sufficient *reversible window* is preserved to support deep tissue reconstruction and the maintenance of regeneration (Salo and Myllyharju, 2021). If the temporal sequence of synthesis-degradation-crosslinking becomes skewed, ECM plasticity plummets, leading to restricted tissue-level reconstruction; thus, even if stem cells remain detectable, lineage output may be chronically deficient. During follicular anagen, downward invagination and tissue reconstruction necessitate that the local ECM possess controlled plasticity within a specific spatiotemporal window. At this juncture, the protease system (e.g., the MMP/TIMP balance) dictates the degradation and renewal window (Kazemi et al., 2025). However, in miniaturizing alopecia, this transient physiological pulse is superseded by a pathological chronic baseline. Persistent low-grade inflammation and oxidative stress disrupt the spatiotemporal equilibrium of MMPs/TIMPs, subjecting the dermal ECM to the dual insults of “chaotic degradation” and “aberrant crosslinking (e.g., AGE-mediated).“ (Wang et al., 2024) This renders the matrix structurally and mechanically resistant to remodeling, ultimately sealing the physiological “plasticity window” and trapping the niche in a rigid, degradation-resistant pathological steady state. This imposes a continuous physical constraint, accelerating anagen truncation and the fixation of superficial, miniaturized structures (Pehrsson et al., 2021). Miniaturized zones exhibit distinct readouts of imbalanced remodeling dynamics that match the temporal scale of the “closed regenerative window”: early stages are more likely to manifest as functional window biases, whereas late stages culminate in irreversible structural locking (Plante et al., 2023) (Table 2).

**TABLE 1:** Five elements of collagen-network-defined niche identity in the hair follicle: operational definitions, measurable readouts, and testable predictions.

Element	Operational definition	Key collagen/ECM structures	Measurable readouts	Representative methods	Falsifiable prediction
Boundary integrity	BM-defined compartmental boundary that confines HFSCs and buffers inflammatory/pro-differentiation cues	Laminin-511/332; Type IV collagen (COL4); BM linkers (nidogen/perlecan)	BM continuity/rupture; thickness/lamination; diffusional barrier proxy; immune cell proximity to bulge	IF/whole-mount; EM; SHG/2P at BM; tracer diffusion assays	BM discontinuity/thickening should co-localize with reduced progenitor markers. If progenitor loss occurs without local BM abnormality, this element is weakened
Adhesion and anchoring	Integrin–BM/hemidesmosome anchorage that retains HFSCs and gates activation–migration cycles	Integrins (alpha6beta4, beta1); hemidesmosomes; COL17A1; anchoring collagens (e.g., COL7)	Hemidesmosome density/continuity; integrin clustering; anoikis resistance; positional stability of HFSCs	IF for integrin/COL17A1; super-resolution; traction/adhesion assays; lineage tracing	Reduced COL17A1/hemidesmosome continuity should predict poor progenitor output better than HFSC abundance alone
Topological organization	Fibrillar collagen geometry (alignment, connectivity, pore size/gradients) setting geometric boundary conditions for polarity and migration	Fibrillar collagens I/III/V; collagen bundles around dermal sheath/DP; interfibrillar ECM components	Anisotropy/alignment order; network connectivity; pore architecture; density gradients; nanoscale topography	SHG/2P; fiber-orientation analysis; 3D imaging + computational topology metrics	SHG-derived alignment/connectivity should distinguish follicles with similar collagen abundance but different output
Mechanical set-point	Homeostatic biomechanical range (elasticity, viscoelastic relaxation, tissue tension) that sets mechanotransduction thresholds	Collagen bundle thickness/crosslinking; PCM components (e.g., COL6); actomyosin–adhesion coupling	Young’s modulus; stress-relaxation/creep; residual tension; YAP/TAZ activation threshold	AFM indentation; micro-rheology; traction force microscopy; YAP/TAZ nuclear localization	Higher stiffness or slower stress relaxation should predict weaker progenitor expansion, reduced follicle depth, or shorter anagen persistence
Dynamic remodeling	Time-windowed balance of synthesis, proteolysis, and crosslinking across the hair cycle that preserves ECM plasticity for reconstruction	MMP/TIMP axis; LOX/AGE crosslinking; collagen turnover machinery	Turnover rate; MMP/TIMP ratio; crosslink burden; reopening of a ‘plasticity window’ during anagen	Zymography; protease panels; crosslink assays; longitudinal imaging across cycle	Higher crosslink burden or persistent MMP/TIMP imbalance should predict poorer durability after growth-signal withdrawal

**TABLE 2:** Evidence stratification for the five elements of niche identity.

Element	Main supporting evidence	Dominant evidence type	Supports correlation, necessity, or sufficiency?	Strongest model context	Main limitation
Boundary integrity	Altered BM continuity, thickness, and compartmental disorganization co-localize with miniaturization, inflammatory overlap, and impaired output	Predominantly correlative/spatially associative	Mainly correlation	Human scalp histology/imaging	Direct boundary-specific perturbation evidence is lacking; necessity and sufficiency remain unresolved
Adhesion and anchoring	COL17A1 loss and hemidesmosome destabilization impair HFSC residence and promote elimination-like phenotypes	Interventional, especially loss-of-function	Strongest support for necessity; sufficiency less established	Murine genetic models	Direct causal relevance to human AGA remains inferential; human rescue/gain-of-function evidence is limited
Topological organization	SHG-defined alignment, connectivity, and pore alterations associate with reduced reconstruction efficiency and miniaturization-like architecture	Predominantly correlative, with mechanistic plausibility from ECM-guidance literature	Mainly correlation	Human imaging + inference from ECM biology	Topology-specific perturbation studies in hair follicles are scarce; necessity/sufficiency not directly established
Mechanical set-point	Niche stiffening, altered viscoelasticity, and mechanotransduction changes modulate regenerative competence; synthetic or engineered matrices can rescue or redirect fate	Interventional/mechanobiological, including controlled perturbation	Supports causal relevance; partial support for sufficiency in engineered systems	Murine aging models; engineered niches; biomaterials systems	Human AGA data remain largely associative; universal pathological thresholds are not yet established
Dynamic remodeling	MMP/TIMP imbalance, altered collagen turnover, and crosslink accumulation track impaired plasticity and reduced reset capacity	Mixed: Correlative in humans, mechanistically supportive in models	Mostly correlation; causal plausibility stronger than direct proof	Human temporal association + model systems	Few longitudinal interventions directly show that restoring remodeling kinetics reopens regeneration in human AGA
Integrated five-element state	Coordinated drift across boundary, anchoring, topology, mechanics, and remodeling better explains low-output stability than any single lesion alone	At present mainly conceptual integration + indirect support	Currently supports working hypothesis, not full proof of necessity or sufficiency	Cross-model synthesis	Multi-element perturbation studies directly testing the full framework are still lacking

### 2.2 The collagen network as an integrated constraint carrier: coupling of interface, topology, and mechanics

The dermal collagen network is traditionally conceptualized as a mere structural scaffold. However, relegating it to a passive load-bearing material fails to explain why the identical anatomical scalp region exhibits divergent lineage outputs across different temporal stages, nor does it account for the synchronous drift observed among the five elements of niche identity.

For the hair follicle, the interface (BM and adhesion machinery), the spatial organization of the fibrillar network (alignment, connectivity, pore gradients), and the material state (elasticity, viscoelasticity, crosslinking burden) are not three isolated parameters. Rather, they are concurrently defined within the same integrated collagenous framework and renewed across the hair cycle.

At the interfacial level, this entails the coupling of the BM with the adhesion apparatus (Liu Y. et al., 2022; Matsumura et al., 2016). BM collagens (primarily the Type IV collagen backbone) establish a selective epithelial-mesenchymal demarcation dictated by their continuity, thickness, and ligand presentation density. By physically engaging integrin/hemidesmosome complexes, this boundary structure feeds into adhesion-dependent survival and homeostatic maintenance pathways. *In vivo* tracking using an eGFP-COL4A2 model has unveiled a spatial gradient in COL4A2 turnover that is intimately coupled with BM expansion and epithelial progenitor proliferation/migration, suggesting that the rhythm of interfacial renewal intrinsically constitutes a tunable niche condition (Wuergezhen et al., 2025; Jones et al., 2024).

At the topological level, the spatial organization parameters of the collagen fibrillar network come into play. The assembly patterns of fibrillar collagens, Types I and III, dictate the degree of orientational order, network connectivity, and pore architecture (Bohringer et al., 2023; Ahmed et al., 2021). These factors geometrically constrain the establishment of cellular polarity, the biasing of division planes, and migration/reconstruction trajectories, while simultaneously altering the effective accessibility of soluble factors within the local matrix (He X. et al., 2023). Research demonstrates that even when organ viability is preserved, the remodeling of niche architecture can profoundly alter the spatial distribution of progenitors and organ efficiency (e.g., resulting in shorter generated hair shafts), thereby cementing “topological/structural organization” as an independent niche variable capable of modulating output. In alopecia-related models, two-photon microscopy and Second Harmonic Generation (SHG) imaging provide a robust avenue for the “quantifiable assessment of topological states co-registered with regenerative phenotypes.” For instance, in an AGA mouse model, Two-Photon Excited Fluorescence coupled with SHG imaging was employed for the synchronous readout of hair shaft morphology and collagen fibril signatures, validating the feasibility of non-invasive, stage-specific quantification of the collagen network under pathological contexts (Liu et al., 2024).

At the mechanical level, threshold ranges are set by local elasticity, viscoelasticity, and crosslinking burden (Saraswathibhatla et al., 2023). The scale of collagen fiber bundles and their crosslinking state collectively determine the Young’s modulus and stress-relaxation properties, thereby defining the activation thresholds for mechanotransduction and downstream transcriptional responses. Recent studies reveal that HFSCs can sense mechanical forces via adhesion structures, with Piezo1 interacting directly with E-cadherin. Applying a mechanical pull on the order of ∼20 pN to E-cadherin triggers Piezo1-dependent local calcium signals (calcium flickers) (Wang J. et al., 2025). Conversely, Piezo1 deletion diminishes cumulative calcium influx and impairs the maintenance of quiescence, indicating that mechanical inputs can directly wire into the molecular circuitry governing stem cell quiescence (Yang et al., 2024). At present, validated AGA-specific mechanical thresholds are not available. Evidence from wound-induced hair follicle neogenesis shows that regenerative output can be bounded by measurable stiffness ranges, but this paradigm is biologically distinct from human AGA and should be interpreted only as proof-of-principle. Therefore, values derived from wound models should not be used as quantitative cutoffs for miniaturizing alopecia. In AGA-focused studies, the priority should be to establish follicle-specific stiffness and relaxation ranges that co-register with progenitor output, follicle depth, and shaft caliber (Harn et al., 2021). By contrast, variables such as BM continuity, COL17A1 continuity, and SHG-derived topology are already quantifiable, but their follicle-specific pathological thresholds still require model-specific calibration.

### 2.3 Niche constraints: ECM mechanics and topology dictate lineage bifurcation

Attributing the regenerative attenuation in miniaturization and alopecia solely to intrinsic cellular defects does not consistently align with histological and cytological observations. In AGA, for instance, clinical biopsies reveal that the HFSC pool is not simply depleted; rather, the transit-amplifying progenitor populations directly responsible for lineage output are significantly diminished. This discrepancy suggests that the restrictive bottleneck lies at the conversion phase from stem cells to progenitors, a transition critically dependent on local microenvironmental conditions (Garza et al., 2011; Li et al., 2026).

Mechanical constraints can alter the accessibility thresholds of exogenous signals and modulate transcriptional plasticity. Aging-associated ECM alterations lead to niche stiffening, which suppresses transcription via mechanical stress-related effects and reduces the chromatin accessibility of bivalent promoters, thereby dampening HFSC activation potential (Koester et al., 2021). This indicates that even in the presence of exogenous growth signals, an adverse mechanical background can physically restrict the chromatin and transcriptional response windows, blunting the magnitude of transcription required for HFSC activation. Conversely, alleviating actomyosin contractility and resetting the cellular mechanical state can promote the initiation of hair regeneration in both young and aged mice, proving that this “upward shift in the activation threshold” is not fundamentally irreversible. Cells can sense ECM stiffness through adhesion receptors such as integrins, translating these physical inputs into transcriptional programs via mechanotransduction pathways. This mechanobiological coupling provides a robust theoretical foundation for understanding the aberrant lineage output observed in pathological states (Allan and Chaudhuri, 2025).

At the topological level, the crux lies in the rewriting of geometric boundary conditions: the alignment, connectivity, and porosity of the fibrillar network alter the clustering of adhesion receptors, the establishment of polarity, and the trajectories of migration and reconstruction. Consequently, identical biochemical signal inputs can yield entirely different cellular behavioral outputs depending on the topology (Kim et al., 2024). During the progression of miniaturization, if collagen fibers lose their orientational order, network connectivity weakens, or pore structures become aberrant, the spatial constraints alone can severely impede the downward extension and reconstruction efficiency of progenitor cells, *even without presuming a global shift in biochemical signaling axes*. This topological explanation corroborates the paradoxical hallmark of miniaturization: “detectable HFSCs accompanied by deficient lineage output.” (Hegde et al., 2025)

Beyond topology, the importance of viscoelasticity is supported mainly by mechanobiological proof-of-principle studies rather than follicle-specific AGA data. Synthetic hydrogel systems show that stress relaxation can regulate stem-cell behavior independently of stiffness, but these values were obtained outside the native hair-follicle context. They should therefore be used to justify the inclusion of viscoelasticity as a measurable variable, not as numerical thresholds for AGA. Follicle-specific studies are still needed to define whether stiffness and relaxation kinetics jointly predict progenitor expansion, anagen persistence, and response durability in miniaturizing alopecia (Chaudhuri et al., 2016). Accordingly, the niche-identity model is supported if follicles or engineered niche models matched for HFSC-marker retention, collagen abundance, or biochemical stimulation still diverge in progenitor output or response durability when their modulus–relaxation states differ; it is weakened if these outcomes are fully explained by HFSC abundance, collagen load, or stiffness alone. Recent spatial transcriptomic studies provide a natural platform for directly testing niche-identity predictions, because they can resolve progenitor-marker distribution and ECM-remodeling programs within the same follicular microdomain. In principle, combining spatial transcriptomics with SHG/2-photon imaging or collagen-subtype mapping could directly test whether progenitor depletion and collagen-network deviation are spatially co-registered at single-follicle resolution, rather than merely co-occurring at tissue scale (Charoensuksira et al., 2025).

To avoid treating all supportive observations as epistemically equivalent, the current evidentiary status of each element is summarized below.

### 2.4 Falsifiability and a crucial experimental test

A decisive experiment would be to use *ex vivo* human miniaturizing AGA follicles and compare whether signal-only rescue and signal + structure rescue are biologically equivalent. This should be viewed as an aspirational falsification framework rather than an immediately executable protocol, because current *ex vivo* human follicle systems do not yet faithfully reproduce long-term cycling, withdrawal kinetics, and matrix-remodeling dynamics. Follicles should be pre-selected to have similar HFSC-marker retention and similar baseline collagen abundance, then randomized into four arms: minoxidil/finasteride-mimetic signaling, matrix-softening/anchoring-restoring intervention, and combination treatment. Candidate structure-directed tools could include localized modulation of collagen crosslinking or LOX activity, AGE-crosslink reduction, controlled matrix-softening approaches, BM-supportive laminin/COL4-mimetic substrates, or interventions preserving the COL17A1/integrin–hemidesmosome axis. Before and after intervention, the niche state should be quantified by (1) BM continuity score, (2) COL17A1 continuity/hemidesmosome integrity, (3) SHG-derived fibrillar alignment, and (4) AFM-derived stiffness and stress-relaxation behavior. Readouts should include matrix/progenitor expansion, anagen persistence, follicle depth, and shaft elongation, followed by assessment after withdrawal of the signaling arm. Because validated AGA-specific thresholds are unavailable, “measurable improvement” should be defined within each experimental system as a prespecified change from baseline in BM continuity, COL17A1/hemidesmosome continuity, SHG-derived collagen organization, stiffness/relaxation behavior, and functional output. The model predicts that if structure truly constrains regenerative durability, then follicles in which matrix mechanics and interface continuity are measurably improved should retain output better after signal withdrawal than follicles receiving signal-only stimulation. If, however, output durability is identical in the two groups despite no structural repair, then the niche-identity model would require revision.

## 3 An atlas of functional specialization within the collagen network: from collagen subtypes to niche regulation

### 3.1 Niche anchoring and boundary maintenance: defining “spatial confinement”

A functional follicular niche fundamentally requires stable compartmentalization to chronically confine HFSCs to the bulge and maintain a distinct epithelial-mesenchymal demarcation. This spatial confinement is primarily orchestrated by the tripartite interfacial system: the BM, the adhesion apparatus, and anchoring fibrils. The continuity and compositional heterogeneity of the BM dictate the sharpness of the epithelial-dermal boundary. Integrin/hemidesmosome complexes confer adhesive stability and transduce structural cues into adhesion-dependent survival and homeostatic signals (including anoikis resistance). Concurrently, anchoring fibrils couple the BM to the underlying dermal collagen bundles, fortifying the boundary against destabilization induced by mechanical perturbations and inflammatory stress. The relative immune privilege of the bulge provides a low-perturbation background for long-term maintenance, yet this phenotypic stability is inextricably linked to the integrity of the compartmental boundary (Meyer et al., 2008).

Among BM-associated components, the transmembrane collagen COL17A1 should be viewed not merely as a structural constituent, but as a candidate regulator of anchoring stability. In murine systems, genetic and age-associated loss of COL17A1 supports a necessity-type role for this molecule in maintaining HFSC residence, because COL17A1 depletion promotes stem-cell detachment, transepidermal elimination, and hair loss–like phenotypes (Matsumura et al., 2016). By contrast, in human miniaturizing alopecia, the evidence remains more inferential: what is currently supported is that alterations of the COL17A1-associated anchoring apparatus are consistent with a boundary-destabilization model, rather than that COL17A1 loss has already been proven to be a direct initiating cause of AGA. Thus, the strongest current interpretation is that early anchoring instability represents a mechanistically plausible and testable contributor to the state in which HFSCs remain detectable but their positional stability and lineage output progressively drift. Taken together, the anchoring element is currently supported by the strongest mechanistic evidence at the level of murine loss-of-function, whereas in human miniaturizing alopecia it remains supported mainly by association and mechanistic plausibility rather than direct proof of causality.

Type IV collagen (COL4) forms the reticular backbone of the BM, primarily dictating boundary continuity and the interfacial presentation landscape. Type VII collagen (COL7) forms the anchoring fibrils that stably couple the BM to the subjacent dermal collagen bundles, serving as the critical structural linchpin for boundary-dermal integration and mechanical resilience (Chung and Uitto, 2010). In the advancing zones of miniaturization, the most informative readouts are typically not changes in the gross quantity of a single collagen. Instead, they lie in the co-localized, synergistic aberrations of the COL17A1-associated anchoring apparatus and the COL4/7-associated boundary structures, and how these structural deficits spatially register with clinical endpoints such as progenitor insufficiency and follicular superficialization.

### 3.2 Topological organization and mechano-tuning collagens: setting the “regenerative window”

Fibrillar collagens (Types I, III, and V) constitute the primary load-bearing network of the perifollicular dermis. Type I collagen serves as the principal tensile backbone. In clinical pathology, its alteration frequently manifests as an exacerbated fasciculation and thickening of perifollicular fiber bundles, accompanied by a spatial realignment of fiber orientation. This shift transitions the matrix from a relatively fine, reticular, and dispersible architecture to a denser, more unidirectional, and circumferentially constrictive fascicular structure. Conversely, Type III collagen represents a finer, more reticular fibrillar organization, generally indicative of higher tissue compliance and a broader latitude for remodeling. In the context of AGA, when the Type III/I assembly ratio chronically skews toward thick fasciculation, the local remodeling window becomes increasingly refractory to resetting on a cyclical timescale. This provides the material-level foundation for the upward shift in the threshold required to sustain regeneration (Chen et al., 2025).

Type V collagen governs fibril diameter and uniformity during early fibrillogenesis (Chandrasekaran et al., 2024). When this “parameter-tuning layer” is disrupted, the fibril diameter spectrum becomes highly heterogeneous, fasciculation escapes regulation, and the perifollicular matrix adopts a more irregular, pseudo-cicatricial architecture (Wenstrup et al., 2011). For processes dominated by chronic miniaturization, such as AGA, this perifollicular backdrop of “thicker, tighter, and less relaxable” fiber bundles mechanically dictates that regeneration is harder to sustain, *obviating the need to presuppose a preceding global inversion of biochemical signaling axes* (Wang C. et al., 2025).

Distinct from the macro-fibrillar skeleton dominated by Types I, III, and V, Type VI collagen is heavily concentrated within the pericellular matrix (PCM) (Wang C. et al., 2025). Its function can be conceptualized as modulating the efficiency of mechano-adhesive coupling between the cell and a stiffened or densified surrounding matrix. When the coupling fidelity of the PCM layer deteriorates, DP cells and surrounding matrix cells exhibit heightened instability in their responses to tensile, compressive, and relaxational mechanical inputs, even if the macroscopic fiber bundles appear morphologically intact. Experimental studies suggest that COL6 is deposited in the hair follicle and is significantly upregulated following cutaneous wounding. Its role is highly context-dependent: under physiological conditions, *Col6a1* deficiency delays the hair cycle and growth, yet under wound-induced conditions, it paradoxically promotes hair growth (Chen et al., 2015). Such findings substantiate the classification of Type VI collagen as a contextual “threshold tuner,” rather than a unidirectional pro- or anti-regenerative factor (Li T. et al., 2025). Col6a1 knockout provides murine mechanistic evidence for pericellular-matrix control of follicular mechanics, but direct translation to human AGA remains inferential.

### 3.3 Microdomain regulation and locally solidified collagens: from the perifollicular deposition zone to the vascular interface

Within the perifollicular deposition zone of advancing miniaturization, beyond the macro-fasciculation of Types I/III, the microdomain rewriting of fibril surfaces and interfacial sites warrants intense scrutiny (Plante et al., 2023). The FACIT (Fibril-Associated Collagens with Interrupted Triple helices) family, such as COL12A1 and COL14A1, decorates Type I collagen fibrils, regulating inter-bundle coupling and interfibrillar sliding. This mechanism facilitates the transition of the deposition zone from a rearrangeable state to one of localized solidification. In cutaneous repair studies, aberrant levels of COL12 have been correlated with altered growth factor bioavailability and skewed immune cell profiling in the wound bed, implying that even minute quantities of these structural molecules can pivot the trajectory of tissue remodeling (Schonborn et al., 2020). Furthermore, COMP (Cartilage Oligomeric Matrix Protein) can concurrently bind COL12 and COL14, enriching at specific structural hubs such as focal adhesions (Agarwal et al., 2012). Consequently, in AGA, the patchy, plaque-like expansion of FACIT/COMP within the perifollicular deposition zone, and their spatial co-localization with immune cell infiltrates or myofibroblast foci, deserves prioritized attention.

Miniaturization is not an exclusively follicular epithelial affliction. The perifollicular ECM “sleeving” or encapsulation concurrently impairs microvascular permeability and the paracrine exchange between the DP and the epithelium, thereby amplifying the “progenitor insufficiency” phenotype. Multiplexin collagens (COL15A1/COL18A1) are widely distributed across vascular and specialized BM zones, participating in interfacial material properties and vascular wall homeostasis (Tomono et al., 2002). In addition, endostatin, the NC1 cleavage fragment of COL18A1, possesses classical anti-angiogenic effects (Zatterstrom et al., 2000; Marneros and Olsen, 2005). In the advancing zones of miniaturization, if the cleavage or deposition profiles at the BM-vascular interface undergo pathogenic shifts, local vascular support and paracrine supply are critically diminished. Under such conditions, even in the presence of exogenous growth-promoting inputs, the maintenance of anagen may be prematurely throttled at the “supply side,” subsequently entering a mutually reinforcing vicious cycle with the localized solidification of the perifollicular deposition zone (Chen et al., 2025; Sun J. et al., 2025).

## 4 Hair follicle miniaturization: histological phenotypes of niche imbalance and restricted regenerative output

### 4.1 Histological and geometric dimensional assessment of miniaturization

**FIGURE 2:**
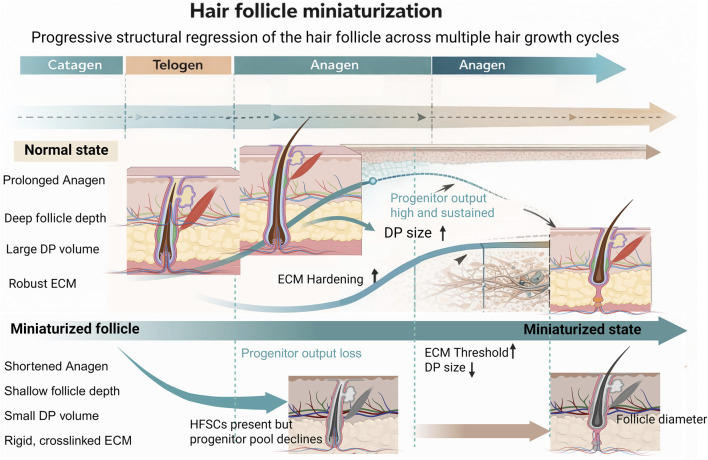
Conceptual trajectory of hair follicle miniaturization across successive hair cycles. Normal state is characterized by a relatively prolonged anagen phase, deeper follicles, a larger dermal papilla (DP), and a more robust extracellular matrix (ECM). In contrast, the miniaturized follicle exhibits a shortened anagen phase, a more superficial follicle, reduced DP volume, and a rigid, crosslinked ECM, leading to persistently diminished regenerative output and progressive follicle downsizing.

Hair follicle miniaturization refers to the progressive transformation of terminal follicles into “vellus-like” follicles over successive hair cycles. Histologically, its essence is not the outright disappearance of the follicular unit, but rather a dimensional reduction and truncated growth phase: the hair bulb and matrix cell zones shrink, the DP volume/cell count decreases, and the follicle as a whole becomes more superficial, producing hair shafts with smaller diameters, lighter pigmentation, and an incomplete medulla (Sinclair et al., 2003; Rushton et al., 2022). In both clinical and research contexts, the most explanatory metric is not merely the average hair diameter, but the width and heterogeneity of the hair shaft diameter distribution. The hallmark of miniaturization is the coexistence of thick and thin hair shafts within the same region, accompanied by a rising proportion of fine hairs (a leftward shift and broadening of the diameter distribution). In trichoscopy, this correlates with increased “hair diameter diversity/variability” and a higher percentage of vellus-like hairs, representing one of the most ubiquitous microscopic features of AGA (Figure 2) (Kasumagic-Halilovic, 2021; Kuczara et al., 2024).

In histological quantification, the terminal-to-vellus (or vellus-like) hair ratio (T:V) serves as a core parameter for diagnosis and subtyping, particularly in horizontal sections, which facilitate easier counting and morphometry. For the differential diagnosis of female pattern hair loss/diffuse thinning, a T:V ratio of <4:1 is commonly employed as a suggestive diagnostic threshold, whereas a ratio >8:1 leans heavily toward non-miniaturizing alopecias, such as chronic telogen effluvium (Sinclair et al., 2004; Bhat et al., 2020; Kibar et al., 2014). It is crucial to emphasize that in research settings, “vellus-like” is often further defined by specific diameter thresholds (e.g., ≤0.03 mm), enabling the mutual calibration between “diameter distribution” and “T:V ratio”. Miniaturization manifests not only as hair shaft thinning but also as the geometric scaling-down of the follicular unit itself: shallower follicular depth and reduced bulb/papilla volume. Consistently, animal models reveal a reproducible correlation between DP cell number and hair shaft size, indicating that DP volume can serve as an indirect readout of “output capacity” (Chi et al., 2013) (Table 3).

**TABLE 3:** Quantifiable readouts of follicle miniaturization across histology and trichoscopy: definitions, thresholds, and interpretive notes.

Metric	Specimen/modality	How to quantify (operational)	Typical interpretation/thresholds	Key confounders/notes
Hair shaft diameter distribution	Trichoscopy; phototrichogram; horizontal sections	Measure many shafts per region; report mean/median and heterogeneity (e.g., SD, CV); track left-shift over time	Progressive left-shift and increased heterogeneity reflect miniaturization progression	Region-specific sampling; hair cycle stage; measurement bias from shaft curvature/cut angle
Terminal-to-vellus(-like) ratio (T:V)	Horizontal scalp sections (gold standard); sometimes trichoscopy proxies	Count terminal vs. vellus-like follicles/shafts in standardized area; compute ratio	Lower T:V indicates patterned miniaturization; thresholds depend on definition used in the study	Definition of vellus-like varies; section depth and orientation strongly affect counts
Vellus-like cutoff	Histology/trichoscopy	Use study-defined diameter cutoff (e.g., ≤ 30 μm) and/or shallow follicle depth; classify vellus-like units	Rising vellus-like fraction indicates transition toward low-output steady state	Cutoff is method-dependent; mixed terminal/miniaturized units complicate classification
Follicle depth/superficialization	Vertical sections 3D imaging	Measure distance from epidermis to bulb/DP; compare affected vs. unaffected regions	Reduced depth correlates with weaker downward reconstruction and shortened anagen	Section plane; tissue shrinkage; anagen substages
Bulb/DP volume and DP cell number (proxy)	Histology; whole-mount; 3D confocal	Segment bulb/DP area or volume; optionally count DP nuclei (DAPI) per unit	Lower bulb/DP size aligns with reduced inductive capacity and thinner shafts	DP boundaries can be ambiguous; cell counting requires consistent markers
Anagen maintenance/A:T ratio	Histology; trichogram	Score anagen vs. telogen follicles; compute anagen:telogen (A:T) or anagen fraction	Prolonged telogen and reduced anagen fraction indicate reduced regenerative flux	Seasonal effects; depilation/handling; strain/sex differences in mice
Perifollicular fibrosis/collagen organization score	Masson’s trichrome; picrosirius red; SHG imaging	Semi-quantitative scoring of collagen thickening; SHG-derived anisotropy/alignment metrics	Higher fibrosis/alignment toward circumferential bundles associates with stiffening and lower output	Scoring subjectivity; need blinded scoring and standardized ROIs
Local stiffness/viscoelastic relaxation (mechanical set-point)	AFM indentation; micro-rheology; shear rheometry (*ex vivo*)	Report Young’s modulus and relaxation time constants; map spatial heterogeneity around follicles	Upward drift (stiffer, lower relaxation) predicts elevated activation threshold and truncated regeneration	Temperature/hydration; sample thickness; probe geometry; anisotropy of tissue

### 4.2 Stem cell retention amidst restricted lineage outputa

Attributing miniaturization exclusively to complete HFSC loss is not fully consistent with human AGA data, but marker-based retention should not be equated with functional integrity. Garza et al. showed that bulge-enriched HFSC populations, defined by CD200^hi/ITGA6^hi phenotypes, remained detectable in bald scalp, whereas CD34-positive progenitor populations were markedly reduced (Garza et al., 2011). These findings support a dissociation between structural or phenotypic HFSC retention and progenitor output. However, they do not prove that retained HFSCs are functionally normal *in vivo*. Functional exhaustion, epigenetic reprogramming, or reduced responsiveness to activation cues may coexist with, or be induced by, niche remodeling. Therefore, the conversion-defect model should be understood as a combined cell–niche failure rather than evidence that HFSCs are intrinsically intact.

This distinction is important for interpreting miniaturization. The persistence of bulge architecture or HFSC-associated markers indicates that the follicle has not undergone complete stem-cell compartment loss, but it does not establish preserved regenerative competence. During disease progression, both HFSC functional capacity and transfer efficiency into the amplifiable progenitor pool may decline. The resulting phenotype is therefore better described as restricted lineage output arising from interacting stem-cell-intrinsic and niche-derived constraints, leading to progressive shaft thinning, follicular superficialization, and a low-output state over successive cycles.

### 4.3 A multidimensional model of miniaturization degeneration

During the progression of miniaturization, one of the most reproducible structural alterations is the progressive thickening and fibrotic “sleeving” of the perifollicular connective tissue sheath. This is characterized by increased collagen deposition, thicker and denser fiber bundles, reduced inter-bundle porosity, and enhanced circumferential constraint. Such alterations have been repeatedly described in histological studies of AGA and are considered to occur in tandem with micro-inflammation/fibrotic processes (El-Domyati et al., 2009; Yoo et al., 2006). Furthermore, research has directly documented a correlation between bulge-region fibrosis and the staging of follicular miniaturization, establishing a quantifiable correspondence between “boundary/perifollicular sleeve structural changes” and the “degree of miniaturization” (Li et al., 2022).

Structural “thickening/densification” is not merely a morphological phenomenon. Enhanced fiber fasciculation and increased crosslinking burden elevate local stiffness, causing the tissue to relax more slowly and dissipate energy less efficiently, thereby trapping cells in an increasingly refractory mechanical background that is harder to “rebound” from (Ahmed and Ffrench-Constant, 2016). In AGA, perifollicular fibrosis is recognized as a critical driver of miniaturization (Li L. et al., 2025). ECM stiffness and cellular geometry can influence cellular transcriptional states and fate thresholds via mechanotransduction axes such as YAP/TAZ (Nam et al., 2025). In chronic remodeling associated with miniaturization, the more pivotal shift is the decline in “remodeling reversibility”: the temporal relationship among degradation, synthesis, and crosslinking becomes biased, resulting in cumulative deposition while making it increasingly difficult to revert to a resetteable window (Hou et al., 2016; Chermnykh et al., 2018). Consequently, the visible histological readout is not just “increased collagen,” but the solidification of remodeling dynamics into a persistent fibrotic background. This works in concert with shortened anagen phases, superficialization, and dimensional scaling-down to drive the low-output steady state.

### 4.4 The miniaturization spectrum across alopecia subtypes: shared endpoints and heterogeneous pathways

The core premise of the miniaturization spectrum is that the HFSC is physically retained, but its lineage output to progenitors is restricted. Both AGA and senescence-associated thinning fit this model seamlessly; their niches reside in a state of reversible or semi-reversible *pathological dormancy*.

In AGA, perifollicular fibrosis runs parallel to chronic low-grade inflammation and fibroblast activation, the severity of which may correlate with clinical refractoriness and insufficient treatment responses. Dihydrotestosterone (DHT) binding to the androgen receptor (AR) in the DP triggers the release of inhibitory factors (such as TGF-β1 and DKK-1) (Kwack et al., 2008). This initiates a paracrine loop that stimulates dermal sheath fibroblasts to deposit excessive Type I collagen, leading to perifollicular fibrosis. In bald scalps, atomic force microscopy and histological analyses reveal that due to collagen fasciculation, the stiffness of the dermal sheath is significantly higher than in non-bald areas (Higgins et al., 2013). This “stiffening” mechanically constrains the follicle, physically impeding the downward invagination required during anagen and effectively trapping the follicle in a miniaturized state. Biopsies frequently reveal “micro-inflammatory” clues such as perifollicular inflammation and follicular spongiosis, indicating a microenvironment characterized by inflammation-fibrosis crosstalk (Plante et al., 2022).

Senescence-associated thinning, distinct from the active suppression seen in AGA, is characterized by an elevated threshold for regenerative initiation, delayed cyclical reentry, and blunted responses to pro-growth inputs. Matsumura et al. discovered that DNA damage responses within HFSCs trigger the proteolysis of COL17A1 (Type XVII collagen), causing stem cells to lose polarity and undergo mechanical delamination (Matsumura et al., 2016). Groundbreaking lineage-tracing studies in aged mice demonstrated that HFSCs lacking COL17A1 lose polarity and undergo symmetric differentiation into epidermal keratinocytes, rather than self-renewing. These cells are mechanically extricated from the niche and cleared via the skin surface, a process termed “stem cell exhaustion via transepidermal elimination” (Keyes et al., 2013). Concurrently, the aged niche exhibits diminished expression of key activating factors (e.g., Foxp1, FGF), implying that a much stronger stimulus is required to trigger regeneration.

Cicatricial (scarring) alopecia serves as the negative control/boundary condition for this framework. Its core pathology is immune attack and structural destruction of the bulge region, resulting in the replacement of the follicular unit by fibrous tissue and its permanent loss. Therefore, a reversible miniaturization process does not exist here; the niche is structurally obliterated. For instance, in Lichen Planopilaris (LPP), the BM zone of lesional follicles displays interrupted expression of Types IV and VII collagen and aberrant morphologies, linking boundary apparatus failure to abnormal repair and scar formation (Al-Refu and Goodfield, 2009). In Frontal Fibrosing Alopecia (FFA), pathology reveals occlusion of the sebaceous glands and bulge region, replaced by concentric lamellar fibrosis. The loss of PPAR-γ is believed to be associated with the metabolic regulation necessary for sebaceous gland and stem cell survival (Karnik et al., 2009). Consequently, when the boundary completely collapses and dense scarring ensues, discussing “topology-mechanical set-point tuning” is insufficient to explain the irreversible outcome. In such scenarios, therapeutic priorities must pivot toward immune control and structural reconstruction. To distinguish shared miniaturization endpoints from distinct pathogenic states, a simplified stratification framework integrating trichoscopy, collagen-network signatures, and predicted therapeutic responsiveness is proposed (Table 4).

**TABLE 4:** Stratified discrimination of AGA, senescence-associated thinning, and fibroinflammatory/scarring alopecia.

Condition	Diagnostic trichoscopy features	Collagen-network/SHG signatures	Predicted therapeutic responsiveness
AGA	Hair shaft diameter diversity, increased vellus-like hairs, reduced terminal-to-vellus ratio, preserved follicular openings, occasional peripilar/perifollicular signs	Perifollicular collagen thickening, increased SHG signal and alignment, reduced pore permissiveness, local stiffening with upward-shifted mechanical set-point	Best suited for niche-targeted + pro-growth strategies; anti-inflammatory therapy may help when microinflammation is active, but is rarely sufficient alone
Senescence-associated thinning	Diffuse density/caliber reduction, usually less marked anisotrichosis and weaker inflammatory signs than AGA	Matrix fragmentation plus stiffening, reduced remodeling plasticity, impaired viscoelastic relaxation, partial loss of reset capacity	More likely to benefit from mechanical/niche-resetting approaches than from broad anti-inflammatory therapy, unless inflammation is superimposed
Fibroinflammatory/scarring alopecia (e.g., FFA/LPP)	Loss of follicular openings, perifollicular erythema/scale in active lesions, scarring background, reduced visible follicular units	Dense scar-like collagen, marked interface disruption, loss of normal follicular architecture, structural replacement rather than reversible drift	Primarily responsive to anti-inflammatory/immune-suppressive therapy in active stages; niche-targeted or pro-growth therapy alone is unlikely to restore durable output once structural destruction is established

The present framework is currently best supported in male AGA. Although FPHL shares the endpoint of follicular miniaturization, its niche-identity profile may differ in at least three respects. First, estrogenic signaling can modulate dermal collagen turnover, fibroblast activity, and MMP/TIMP balance, suggesting that the remodeling-window element may be hormonally more dynamic in FPHL. Second, menstrual or menopausal endocrine variation may alter ECM degradation, crosslinking, and inflammatory tone, potentially changing the temporal stability of the mechanical set-point. Third, the spatial pattern of FPHL is usually more diffuse and less frontotemporal than male AGA, so boundary, topology, and perifollicular matrix changes may be less regionally polarized. Therefore, the framework should be considered best supported for male-pattern AGA, while its application to FPHL requires sex-stratified validation rather than direct extrapolation.

## 5 Niche vulnerability in hair follicle senescence: shifts in collagen network remodeling

### 5.1 Altered regenerative dynamics: transition to a “high-threshold” state

Before delving into pathological remodeling, it is imperative to recognize that the physiological hair cycle inherently constitutes a dramatic and stringently controlled spatiotemporal ECM remodeling process (Bellani et al., 2025). During the normal catagen-to-anagen transition, local matrix metalloproteinases (MMPs) generate a transient “plasticity window.” This moderately relaxes the matrix to accommodate the downward invagination of the follicle and the volumetric expansion of the DP (Lei et al., 2021). Concurrently, tissue inhibitors of metalloproteinases (TIMPs) precisely terminate this proteolytic phase.

Senescence-associated thinning and miniaturization initially manifest as a global deceleration of hair cycle dynamics. Prolonged telogen phases, delayed anagen reentry, and compromised anagen maintenance ultimately culminate in a decline in regenerative flux per unit time (Bellani et al., 2025). During this phase, the absolute number of HFSCs does not necessarily decline commensurately with the macroscopic phenotype. A more consistent alteration is the blunted responsiveness of HFSCs to initiating signals, effectively raising the “activation energy” required to launch a new cycle. Research reveals that age-associated ECM stiffening and altered mechanical landscapes parallel a decrease in HFSC chromatin accessibility (Lee and Choi, 2024). This specifically impacts key self-renewal and differentiation loci marked by bivalent modifications, thereby eroding the transcriptional plasticity required for regeneration. Placing these aged HFSCs into a controlled synthetic niche partially rescues these defects, indicating a quintessential “extrinsic constraint-driven” aging phenotype.

This sluggishness is not inherently cell-autonomous; rather, it is dictated by the aged collagen network, which fails to efficiently relay indispensable “awakening” signals (e.g., Wnt/β-catenin) while simultaneously accumulating inhibitory cues (e.g., BMPs). Consequently, the follicle enters a state of “stalled quiescence,” unable to overcome the biophysical inertia of the aged microenvironment. Correspondingly, if the mechanical background is reversibly modulated, regeneration can be re-initiated. In mouse skin, miR-205-mediated modulation of actomyosin tension is sufficient to re-initiate regenerative behavior, thereby providing gain-of-function evidence that mechanical state can influence follicular output. However, this should be interpreted as murine sufficiency-type support for mechanoregulation, not as direct therapeutic proof in human AGA.

### 5.2 COL17A1 and hemidesmosomes: niche interfacial nodes and the “reversible boundary”

The causal link between COL17A1 depletion and follicular senescence/miniaturization provides compelling evidence that “interfacial apparatuses are regenerative rate-limiting variables” (Nanba et al., 2021). As a transmembrane collagen and a core component of hemidesmosomes, COL17A1 tethers HFSCs to the BM, forming a structural unit capable of bearing tension and transducing adhesion-dependent survival and quiescence signals (Liu et al., 2019; Nishie, 2020). The COL17A1-hemidesmosome axis operates as an intrinsic “niche component” during skin homeostasis, aging, and repair, exerting its effects primarily by modulating stem cell adhesion, localization, and fate bias (Liu Y. et al., 2022; Matsumura et al., 2016). The “hemidesmosome-type adhesion units” within the follicle are regarded as foundational architectures for epithelial-ECM cross-talk, dictating the mode of mechano-biochemical coupling during regeneration (Watanabe et al., 2017).

DNA damage and oxidative stress within aged stem cells trigger the specific proteolysis of COL17A1 by neutrophil elastase or MMPs. The degradation of COL17A1 precipitates the disassembly of hemidesmosomes. Bereft of this physical tether, HFSCs lose their apical-basal polarity and subsequently undergo symmetric differentiation into epidermal keratinocytes, forgoing self-renewal. This phenomenon, termed “stem cell exhaustion via trans-epidermal elimination,” acts as a direct driver of the miniaturized phenotype.

### 5.3 The dual perils of the aged collagen network: fragmentation vs. stiffening

The aged ECM does not undergo a unidirectional “depletion.” More commonly, two modes of physical failure coexist across different spatial scales. The first is structural disconnection (fragmentation) at critical interfaces, manifesting as the thinning or loosening of the ECM at the DP-hair matrix interface, the PCM, or the BM, thereby dampening epithelial-mesenchymal coupling efficiency (Mayorca-Guiliani et al., 2025). For instance, studies using multimodal imaging have directly visualized the profound depletion of the ECM within the DP of aged follicles, proposing that the downregulation of THSD4 enrichment at the DP-HM interface directly correlates with attenuated senescent cross-talk (Wang M. et al., 2025).

The second mode is material-level mechanical locking. Here, the crosslinking burden of the residual matrix escalates while its viscoelastic stress-relaxation capacity diminishes, rendering the tissue stubbornly resistant to reverting to the pliable state required for regeneration, even in the presence of exogenous signals. Consequently, the senescent follicular niche is structurally “disconnected” (impeding directional cell migration and signal diffusion) while simultaneously being mechanically “stiffened” (obstructing follicular volumetric expansion and physical remodeling) (Asgari et al., 2026). This dual failure mode of concurrent fragmentation and stiffening provides a biophysical explanation for why the solitary administration of pro-growth factors often fails to rejuvenate aged follicles (specific pathogenic pathways are detailed in Sections 6.1 and 6.2).

### 5.4 The regenerative window and niche resilience

The regenerative window of the aging follicle serves as a composite readout of niche resilience. “Niche resilience”-the capacity of the ECM to recover its plastic state-is the ultimate determinant of reversibility (Wu et al., 2025). The integrity of the interface hinges on the continuity and thickness of the BM, alongside whether anchoring apparatuses (typified by the COL17A1-hemidesmosome complex) retain structural continuity and the capacity for reconstruction. Conversely, the core determinant of the mechanical set-point is whether local stiffness and viscoelastic relaxation properties have drifted upwards and become refractory to resetting, thereby systemically elevating the threshold for sustaining regeneration despite the presence of exogenous signals (Lennon and Sherwood, 2025). In this context, regenerative capacity is determined not by stiffness alone, but also by relaxation kinetics; specifically, whether the local matrix retains a resettable relaxation timescale is critical for preserving a reversible regenerative window. Supporting this concept, recent *in vivo* work in naturally aged skin shows that red-light photobiomodulation can partially reverse age-related collagen stiffening through TGFβ/AKT-mediated collagen dynamics, providing a concrete example that matrix hardening can remain intervention-responsive rather than irreversibly fixed (Chang et al., 2025).

Furthermore, remodeling reversibility dictates whether the temporal dynamics of synthesis, degradation, and crosslinking can still revert to a plastic phase within the timescale of a hair cycle. A state characterized by an intact interfacial apparatus, a stable mechanical set-point, and preserved reversible remodeling windows signifies *recoverable remodeling*. In stark contrast, when loosened or shattered interfacial anchoring coalesces with an upward-shifted set-point and chronic deposition or crosslinking lock-in, the niche adopts a “*pathologically homeostatic” phenotype* (Sloseris and Forde, 2025). This manifests as compromised resilience and a narrowed regenerative window, wherein therapeutic interventions are prone to yielding only transient or unstable regenerative gains (Liu Y. et al., 2022; Sun Y. et al., 2025). These data provide strong mechanistic support in murine or engineered systems, but their direct translational equivalence to human AGA should not be assumed.

## 6 Diverse etiologic inputs converge on collagen networks to re-specify niche identity

### 6.1 The degradation axis: oxidative stress and MMP-Mediated loss of connectivity

Chronic oxidative stress (e.g., photoaging or metabolic senescence) drives the accumulation of reactive oxygen species (ROS), which persistently activate MMP-1 and MMP-3 via the AP-1/NF-κB signaling axis (Hou et al., 2016; Du et al., 2024). This shift of MMP activity from a physiological transient pulse to a pathological chronic baseline acts as the core driver for the aforementioned “interfacial disconnection.” Distinct from the stringently controlled, spatially and temporally targeted proteolysis during a healthy hair cycle (such as MMP-9 activity at the anagen hair bulb), this unbridled chronic hydrolysis results in the stochastic fragmentation of fibrillar collagens (Types I and III) (Corominas-Murtra et al., 2020).

As demonstrated by Fisher et al. in skin aging models, fragmented collagen fails to provide the requisite mechanical tension for fibroblast spreading. Consequently, fibroblasts collapse, precipitating a reduction in *de novo* collagen synthesis and a reciprocal upregulation in MMP secretion, thereby instigating a self-perpetuating vicious cycle of matrix degradation (Fisher et al., 2009). Within the follicular network, this loss of topological connectivity structurally severs the signal cross-talk pathways between the DP and the bulge region. Deprived of a continuous ECM to serve as an effective diffusional medium, the spatial reach of critical biochemical gradients (e.g., Wnt) is severely curtailed. Consequently, although stem cells remain positionally retained, they are unable to initiate regenerative programs due to this microenvironmental signal “disconnection” (Keyes et al., 2013; Corominas-Murtra et al., 2020; Rompolas et al., 2012).

### 6.2 The crosslinking and stiffening axis: glycation and “mechanical locking”

While MMP-mediated degradation severs network connectivity, the accumulation of non-enzymatic crosslinks driven by metabolic dysfunction and aging further stiffens the residual matrix. This axis elucidates the “irreversibility” frequently observed in chronic alopecia. Advanced glycation end-products (AGEs, such as CML and pentosidine) form pervasive crosslinks between collagen fibrils, rendering them brittle and rigid (Wang et al., 2024; Chen et al., 2022). Unlike physiological enzymatic crosslinking mediated by lysyl oxidase (LOX), glycation-induced crosslinks are profoundly stable and highly resistant to MMP degradation.

The pathological crux lies in the cumulative and renewal-resistant nature of this crosslinking burden. As the crosslinking load escalates, the network, even when degraded, tends to persist as a “fragmented yet stiff” structure. This precipitates “remodeling hysteresis”, a state where the ECM fails to revert to the plastic phase required to accommodate volumetric expansion, migration, and tissue reconstruction within the timescale of a hair cycle. More critically, this aberrant upward shift in the mechanical set-point translates directly into intra-nuclear transcriptional suppression (Vivo et al., 2024). Koester et al. unequivocally demonstrated that niche stiffening, via mechanotransduction effects, reduces the chromatin accessibility of key initiating genes (particularly promoters bearing bivalent modifications), thereby systemically dampening the amplitude of the transcriptional response required for stem cell activation (Koester et al., 2021). Consequently, even upon the reception of regenerative signals, the physical matrix remains too rigid to expand, forcing stem cells into a stalled state. Furthermore, glycation-induced mechanical stiffening frequently synergizes with oxidative stress, driving the senescence and apoptosis of DP cells, which ultimately depletes the follicular inductive core (Ru et al., 2025).

### 6.3 The fibrosis axis: chronic stimulation and the “pseudo-scarring” niche

Chronic stimulation drives localized fibrotic responses, creating a hostile microenvironment akin to scar tissue. Sustained androgenic stimulation triggers the release of TGF-β1 from DPCs, acting on surrounding dermal sheath fibroblasts to induce their differentiation into myofibroblasts (Inui et al., 2002). These activated cells deposit dense, disorganized, and hyper-fasciculated Type I collagen bundles, histologically identified as “fibrous streamers” subjacent to miniaturized follicles.

In AGA, the more prevalent morphology of “perifollicular fibrosis” is not the rigid, concentric lamellar scarring characteristic of classical cicatricial alopecia. Rather, it manifests as mild-to-moderate peripilar collagen thickening around the infundibulo-isthmic region, frequently accompanied by α-SMA-positive interstitial cells indicative of localized myofibroblast-like activation. In studies focusing on PIILIF (perifollicular infundibulo-isthmic lymphocytic infiltrate and fibrosis), this combination of “inflammation plus mild fibrosis” is observed in a substantial proportion of pattern hair loss specimens, whereas “overt concentric lamellar scarring” remains uncommon. This suggests that fibrosis participates in shaping the miniaturizing environment primarily via chronic thickening and localized solidification (Valdebran et al., 2020). Correspondingly, comparative analyses using horizontal sections of terminal versus miniaturized follicles reveal that micro-inflammatory phenotypes (e.g., perifollicular inflammatory infiltrates, follicular spongiosis) can be more pronounced in miniaturized follicles. This pathologically substantiates that inflammatory activity is concurrent with miniaturization, debunking the notion that fibrosis is merely an end-stage depositional consequence (Plante et al., 2022). The microinflammatory background accompanying perifollicular fibrosis is unlikely to be a passive bystander, because its fibrogenic effect is probably executed through specific immune–stroma circuits (Mavrogonatou et al., 2023; Pamonag et al., 2022). In this setting, macrophages are not a uniform population: pro-fibrotic polarization states can reinforce fibroblast activation through TGF-β-rich and matrix-remodeling secretomes, whereas persistent inflammatory polarization may sustain protease imbalance and aberrant ECM turnover (Jiang et al., 2024). Type-2 cytokines, particularly IL-4 and IL-13, can promote dermal fibroblast activation and collagen deposition, supporting the broader concept that inflammatory cytokine milieus may bias perifollicular fibroblasts toward ECM accumulation and remodeling deviation (Nguyen et al., 2020). IL-17 enhances collagen synthesis/fibrosis-associated programs, whereas IFNγ reshapes collagen matrix deposition and remodeling behavior in a context-dependent manner (Park et al., 2018).

Recent dermatopathological investigations note the presence of “perifollicular microscarring” in biopsies of pattern hair loss, a feature not exclusive to scarring alopecia but also emergent within certain non-scarring alopecia contexts. The ECM alterations in AGA can present as micro-scale collagenous thickening and structural rearrangement around the follicle, trending toward a “pseudo-cicatricial solidification.” However, this trajectory typically does not culminate in the complete replacement of the follicular unit by fibrous tissue (Merlotto et al., 2020). The TGF-β/Smad-dominated fibrotic program undergoes crosstalk with regeneration-associated pathways (e.g., Wnt/β-catenin, Notch), chronically eroding epithelial-mesenchymal communication efficiency and elevating the threshold required to sustain regeneration (Gumede et al., 2024). Consequently, perifollicular fibrosis and miniaturization advance in a parallel, mutually reinforcing manner (Wang et al., 2022).

### 6.4 A unified model: tripartite instability synergistically alters niche identity

Despite the intrinsic resilience of the hair follicle, its terminal exhaustion is not the consequence of a singular defect. Rather, it is the product of the synergistic interplay among matrix degradation, collagen crosslinking, and BM instability (Mavrogonatou et al., 2023). MMP-mediated proteolysis fragments fibrillar collagens, shattering the structural continuity of the dermis. This obliterates the contact guidance cues essential for the directional migration and orientation of progenitor cells, resulting in a disorganized growth vector (Pamonag et al., 2022). Concurrently, the accumulation of AGEs induces non-enzymatic crosslinking, significantly elevating the elastic modulus (stiffness) of the perifollicular dermis (Kamml et al., 2023). This imposes a compressive physical constraint, wherein the mechanical resistance of the matrix supersedes the turgor pressure generated by proliferating cells, mechanically stifling the downward expansion of the hair germ. Furthermore, the targeted proteolysis of COL17A1 dismantles hemidesmosome integrity. This triggers stem cell delamination from the basal lamina, severing the juxtacrine signaling loops requisite for maintaining stem cell polarity and quiescence. This physically elucidates the paradoxical observation in miniaturized follicles: the preservation of the stem cell pool alongside a precipitous drop in lineage output.

Within this unified model of tripartite instability, collagen network remodeling is not merely a passive downstream byproduct of androgens (DHT) or micro-inflammation; instead, it establishes a pathological positive feedback loop that actively represses regeneration. When early biochemical stimuli trigger initial perifollicular fibrosis and elevated stiffness, this physical constraint actively intervenes in stem cell fate decisions via mechanotransduction pathways (Katoh, 2025). Specifically, matrix stiffening augments cell-surface integrin clustering, activating focal adhesion kinase (FAK) and ramping up cytoskeletal tension, which directly drives the sustained nuclear translocation of the mechanosensitive transcriptional coactivators YAP/TAZ. Within the follicular niche, the aberrant activation of YAP/TAZ not only represses classical anagen-initiating signals (e.g., the Wnt/β-catenin pathway) but also forces stem cells to skew toward epidermal keratinocyte differentiation. Simultaneously, mechanosensitive ion channels like Piezo1 elicit calcium influx in response to abnormal tension, further subverting homeostatic quiescence (Nam et al., 2025).

Ultimately, introducing this physiomechanical perspective resolves the causality dilemma in miniaturization mechanisms: the mechanical locking of the collagen network translates transient upstream pathogenic stimuli into chronic transcriptional repression at the molecular level, thereby cementing its status as the core driver of miniaturization.

## 7 Therapeutics and translation: stratified strategies targeting niche identity repair

Current pharmacological interventions, such as minoxidil and finasteride, predominantly target specific biochemical signaling pathways (potassium channels and androgen receptors, respectively) rather than the structural microenvironment. Although these agents can transiently compel the follicle to re-enter anagen, they generally fail to reverse the underlying collagen remodeling, including chronic fibrosis and matrix stiffening (Messenger and Rundegren, 2004). This discrepancy may contribute to a “signal–structure mismatch,” in which biochemical signals favor follicular growth while the physical niche remains in a biologically restrictive state. Consequently, one limitation of current therapies may be incomplete durability after treatment withdrawal, including recurrent shedding in some patients. We propose that this phenomenon may be partly explained by hysteresis within the collagen network. Improving durability may require moving beyond the induction of hair shaft generation and considering whether components of niche identity can also be restored (Chen et al., 2025).

By “signal-structure mismatch,” we mean a state in which treatment transiently shifts signaling toward regeneration without restoring the niche to a sufficiently permissive structural-mechanical condition. Under this model, visible regrowth may occur during therapy, yet remain externally supported rather than self-sustaining if interface continuity, anchoring stability, topological permissiveness, or matrix reset capacity are not adequately corrected. A quantitative implication is that signaling withdrawal and phenotypic relapse occur on partially separable timescales: after finasteride discontinuation, DHT returns toward baseline within about 14 days, whereas reversal of the hair-count benefit typically unfolds over about 12 months; similarly, recent reviews and consensus guidance indicate that the benefit of minoxidil is maintenance-dependent, with discontinuation followed by progressive loss of the gained effect rather than immediate collapse. This temporal dissociation is consistent with the idea that signal support can change rapidly, whereas niche structure is not repaired on the same timescale. In this sense, durable response requires not complete normalization of all niche variables, but sufficient structural repair that output remains stable even as exogenous signaling is reduced (Ntshingila et al., 2023; Mohy et al., 2025; Nestor et al., 2021). This temporal dissociation can be interpreted as a two-timescale process. Pharmacologic or endocrine signals may rebound within days to weeks after withdrawal, whereas perifollicular matrix remodeling proceeds over hair-cycle timescales. The model therefore predicts delayed relapse: rapid restoration of the upstream pathogenic signal, followed by slower re-accumulation of matrix stiffness, reduced relaxation capacity, anagen shortening, and gradual loss of shaft caliber. A signal-structure “match” would require not only increased hair count during treatment, but also measurable structural stabilization, such as reduced perifollicular collagen thickening, lower SHG-derived collagen alignment/fasciculation, improved follicular depth, and sustained shaft caliber after withdrawal.

### 7.1 Stratified intervention pathways

The parallel presentation of perifollicular inflammation and fibrosis observed in AGA histology suggests that persistent low-grade inflammation is not merely a bystander effect, but a plausible upstream stimulus driving ECM deviation. Therefore, beyond broad-spectrum anti-inflammatories, there is a critical need to precisely target the ROS-MMP loop. In principle, agents that inhibit AP-1 signaling or selectively modulate MMP-1/3 activity could help preserve fibrillar-network connectivity and prevent remodeling dynamics from shifting from cyclical renewal toward a pathological steady state. However, the translational feasibility of MMP-targeted strategies in miniaturizing alopecia remains uncertain. Key unresolved issues include target specificity, pharmacokinetic control, local delivery, and dose-response characterization (Chang, 2023).

Furthermore, JAK inhibitors such as tofacitinib may influence not only cytotoxic T-cell activity in alopecia areata but also immune-stroma crosstalk within the perifollicular niche (Liu et al., 2023; Hou et al., 2022). Their potential relevance to miniaturization would therefore lie in limiting cytokine-driven fibroblast reprogramming and preventing ECM deviation from consolidating into a pro-fibrotic, mechanically restrictive background (Wang et al., 2022). This interpretation is supported mainly by preclinical fibrosis studies and disease-focused reviews indicating that JAK-STAT signaling can contribute to fibroblast activation and matrix remodeling. However, in AGA, these data remain indirect; scalp-specific delivery, dose-response relationships, and durable effects on follicular miniaturization have not yet been established.

Whether the interfacial apparatus remains continuous (encompassing the COL4/laminin BM zone, the basal HFSC α6β4-hemidesmosome complex, and the distribution of bulge COL17A1) directly dictates whether the “presence of stem cells” can successfully translate into sustainable terminal hair output. Once this continuity is compromised, the common outcome is not the immediate obliteration of HFSCs; rather, HFSCs remain detectable but suffer from diminished positional and output stability. This manifests clinically as disconnected progenitor supply chains, a persistent leftward shift in the diameter spectrum, and exacerbated superficialization. The capacity of exogenous pro-growth inputs to yield sustainable terminal hairs hinges on whether this interface remains within a reversible window. If the interface is preserved, stable therapeutic gains are more plausible; if the interface is shattered, merely amplifying pro-growth signals is more likely to yield ephemeral gains followed by rapid relapse. This underscores the translational value of recent explorations centering on COL17A1 in AGA-like models. For instance, apocynin, an NADPH oxidase inhibitor, has been validated to promote COL17A1 stability in keratinocytes, thereby maintaining the stem cell pool and preventing miniaturization in murine models (Liu et al., 2019).

When the perifollicular ECM enters a state of “micro-scale solidification or pseudo-scarring,” merely upregulating growth signals is akin to attempting to move a mechanically locked system. In such scenarios, Controlled Physical Perturbation (e.g., fractional lasers or microneedling) may do more than merely enhance topical permeability; in murine wound-healing contexts; it induces a “Wound-Induced Hair Neogenesis” (WIHN) response. This process has been shown in murine systems to promote the enzymatic breakdown of crosslinked, stiffened scar tissue and to stimulate matrix remodeling, including the deposition of more permissive collagen states, effectively resetting the mechanical set-point to a softer, more permissive state. Along the same logic, photobiomodulation may represent another niche-targeted physical intervention that acts by resetting matrix mechanics rather than merely amplifying pro-growth cues. Recent *in vivo* evidence in naturally aged mice demonstrated that red-light photobiomodulation reverses age-associated collagen stiffening via TGFβ/AKT-dependent collagen remodeling, thereby providing a concrete example that pathological matrix hardening can remain therapeutically reversible (Chang et al., 2025). However, the strongest mechanistic support for these resetting effects still derives from murine or age-related model systems, and their quantitative equivalence in human AGA remains to be established. Thus, these approaches are best interpreted at present as mechanistically supported translational directions rather than clinically validated niche-repair strategies.

### 7.2 Upgrading clinical endpoints: from density to niche quality

To genuinely evaluate the repair of niche identity, clinical assessments should not rely solely on hair density (hairs/cm^2^). As demonstrated by early minoxidil trials, a solitary increase in density often reflects the ephemeral growth of indeterminate, vellus-like hairs rather than robust terminal hair regeneration. Therefore, we advocate for a three-dimensional upgrade of efficacy endpoints. Efficacy should be quantified using parameters such as “Cumulative Hair Thickness” or the “Hair Mass Index (HMI)” rather than simple counts. The hallmark of successful niche repair is a statistically significant rightward shift in the hair shaft diameter histogram (Park et al., 2025). For example, an increase in average diameter from <30 μm to >60 μm signifies the physical volumetric expansion of the hair matrix. The restoration of the terminal-to-vellus (T:V) ratio to >4:1 (Whiting’s histological cutoff for non-bald scalps) serves as the most robust biomarker that the regenerative window has been successfully reopened (English et al., 2022). If a therapy increases density but fails to restore this ratio (remaining <4:1), it indicates a failure to rescue the deeper adipose niche.

Given that fibrosis mechanically locks the follicle in a miniaturized state, we recommend the integration of Optical Coherence Tomography (OCT) and Multiphoton/SHG imaging. SHG can specifically visualize the alignment, density, and crimping of Type I and III collagen bundles without the need for exogenous fluorescent labels. By quantifying these optical signals, clinicians can derive a micro-scale “remodeling index” for the niche, serving as a direct, *in vivo* surrogate endpoint for the successful degradation of restrictive collagen crosslinks.

### 7.3 Risks and modes of failure

When targeting niche identity repair, therapeutic “instability” typically stems from a failure to reset constraint variables, or from treatment-induced collateral damage that paradoxically exacerbates remodeling bias (Kuczara et al., 2024). The most common false-positive readout is an increase in density or anagen ratio without a concomitant rightward shift in diameter distribution or recovery of the T:V ratio. This indicates a purely pro-growth effect rather than a genuine reversal of miniaturization; thus, diameter distribution, T:V ratio, and HMI/cross-sectional area must be designated as primary endpoints with predefined clinical futility thresholds (Pirmez, 2023; Wikramanayake et al., 2012).

Secondly, irritant contact dermatitis and barrier disruption provoked by topical regimens or adjunctive therapies can manifest on trichoscopy as perifollicular erythema and scaling, establishing a mutually amplifying cycle with subsequent fibrotic tendencies (Alajaji, 2024). When procedural treatments (microneedling/energy-based devices) employ inappropriate parameters, controlled micro-injuries can degrade into chronic stress, presenting as prolonged erythema, folliculitis-like papules, or post-inflammatory hyperpigmentation. “Reaction duration” must be established as a hard threshold to promptly de-escalate intensity or pause treatment (Ahmed et al., 2025). Long-term maintenance is further constrained by systemic adverse effects and the collapse of patient compliance; thus, utilizing predefined scales and follow-up checkpoints to report the composition of discontinuation reasons is highly recommended (Abdin et al., 2022). For biologics and PRP therapies, a minimum processing parameter set must be documented to preclude batch-to-batch efficacy fluctuations (Anitua et al., 2025). Finally, against a backdrop of perifollicular stiffening or fibrosis, the introduction of pro-growth inputs may yield transient T:V improvements followed by inevitable relapse. Incorporating co-localized, non-invasive mechanical readouts (e.g., Shear Wave Elastography, SWE) registered with structural endpoints can effectively differentiate transient hyperplasia from sustained phenotypic reversal following the genuine release of mechanical constraints (Lee et al., 2019).

## 8 Conclusion

Hair follicle miniaturization may be understood not only as a consequence of cell-intrinsic dysfunction, but also as a progressive decline in regenerative output associated with altered structural and mechanical constraints within the local niche. In this review, we propose that “Niche Identity,” with the collagen network as a major organizing framework, offers a useful way to integrate boundary integrity, anchoring, topology, mechanics, and remodeling dynamics into a common interpretive model.

Within this perspective, androgen-biased profibrotic remodeling, chronic inflammation-associated matrix degradation, and aging-related crosslinking may converge on a shared outcome: a niche state that becomes less permissive for sustained progenitor output and stable follicular regeneration. Available evidence supports the interpretation that collagen-network remodeling can influence follicular behavior through changes in viscoelasticity, adhesion, and mechanotransduction-associated pathways, including YAP/TAZ- and Piezo1-related responses. However, this framework should be regarded as an integrative and testable model rather than a definitively validated causal hierarchy. A further limitation is that this article is a narrative mechanistic synthesis rather than a systematic review. Because no PRISMA-ScR workflow, predefined search string, or formal inclusion/exclusion criteria were applied, the evidence base should be interpreted as selective and hypothesis-generating rather than exhaustive.

From a translational standpoint, the concept of “signal–structure mismatch” may help explain why biochemical stimulation alone often produces incomplete durability. Future work may therefore benefit from combining pro-growth strategies with approaches aimed at restoring local matrix permissiveness, interfacial integrity, and remodeling plasticity. In this sense, the framework presented here is intended not as a final mechanistic conclusion, but as a literature-based model to guide future experimental testing and therapeutic refinement.
